# Integrin-linked kinase regulates migration and proliferation of human intestinal cells under a fibronectin-dependent mechanism

**DOI:** 10.1002/jcp.21963

**Published:** 2010-02

**Authors:** David Gagné, Jean-François Groulx, Yannick D Benoit, Nuria Basora, Elizabeth Herring, Pierre H Vachon, Jean-François Beaulieu

**Affiliations:** CIHR Team on the Digestive Epithelium, Département d'Anatomie et de Biologie Cellulaire, Université de SherbrookeSherbrooke, Québec, Canada

## Abstract

Integrin-linked kinase (ILK) plays a role in integrin signaling-mediated extracellular matrix (ECM)–cell interactions and also acts as a scaffold protein in functional focal adhesion points. In the present study, we investigated the expression and roles of ILK in human intestinal epithelial cells (IECs) in vivo and in vitro. Herein, we report that ILK and its scaffold-function interacting partners, PINCH-1, α-parvin, and β-parvin, are expressed according to a decreasing gradient from the bottom of the crypt (proliferative/undifferentiated) compartment to the tip of the villus (non-proliferative/differentiated) compartment, closely following the expression pattern of the ECM/basement membrane component fibronectin. The siRNA knockdown of ILK in human IECs caused a loss of PINCH-1, α-parvin, and β-parvin expression, along with a significant decrease in cell proliferation via a loss of cyclin D1 and an increase in p27 and hypophosphorylated pRb expression levels. ILK knockdown severely affected cell spreading, migration, and restitution abilities, which were shown to be directly related to a decrease in fibronectin deposition. All ILK knockdown-induced defects were rescued with exogenously deposited fibronectin. Altogether, our results indicate that ILK performs crucial roles in the control of human intestinal cell and crypt–villus axis homeostasis—especially with regard to basement membrane fibronectin deposition—as well as cell proliferation, spreading, and migration. J. Cell. Physiol. 222: 387–400, 2010. © 2009 Wiley-Liss, Inc.

The integrin-linked kinase (ILK) is typically located in integrin-mediated adhesion complexes formed within the context of extracellular matrix (ECM)–cell interactions (Vouret-Craviari et al., 2004; McDonald et al., 2008). Although its Ser/Thr kinase activity is engaged upon integrin binding, ILK also functions as a scaffolding protein which mediates linkage of integrins to the actin cytoskeleton (Sepulveda and Wu, 2006; McDonald et al., 2008). To this effect, ILK not only directly associates with the β1 integrin subunit but recruits the scaffold-interacting partners PINCH (-1 or -2), as well as one member of the parvin family (α-, β-, or γ-parvin) in order to constitute a PINCH–ILK–parvin (PIP) complex (Legate et al., 2006; Sepulveda and Wu, 2006; McDonald et al., 2008). In addition to creating a physical link between integrins and the actin cytoskeleton, distinct PIP complexes can recruit additional cytoskeletal scaffolding and signaling molecules, such as Nck2, paxillin, α-PIX, and α-actinin (Legate et al., 2006; Sepulveda and Wu, 2006; McDonald et al., 2008). Interactions of PIP complexes with specific additional partners either directly or indirectly activate key elements of various signaling pathways such as Akt and members of the Rho GTPase family, thus implicating ILK in various cell adhesion-mediated processes such as spreading, migration, survival, and proliferation (Legate et al., 2006; Sepulveda and Wu, 2006; McDonald et al., 2008).

The human intestinal epithelium is an elegant biological model for the study of the coordinated cell processes involved in tissue homeostasis. Its rapid, continuous renewal consists of spatially separated proliferative/undifferentiated and non-proliferative/differentiated epithelial cell populations located, respectively, in the crypts and the villi (Pageot et al., 2000; Crosnier et al., 2006). The dynamic renewal of the intestinal epithelium is essentially characterized by intestinal epithelial cell (IEC) production and maturation in the crypts, migration out of the crypts onto the base of the villi and, thereafter, further migration of differentiated cells to the tip of the villi where they are shed by anoïkis (Crosnier et al., 2006). In humans, functionality of the crypt–villus axis is already fully established by mid-gestation (18–20 weeks) (Teller and Beaulieu, 2001; Crosnier et al., 2006). As with all epithelia, the intestinal epithelium interacts with a basement membrane (BM). The intestinal BM directs several cell processes that are critical to the homeostasis of the intestinal epithelium, such as proliferation, migration, differentiation, and survival (Beaulieu, 1997; Simon-Assmann et al., 1998; Teller and Beaulieu, 2001). Some ECM components, such as type IV collagen and the laminins, are exclusive to BMs (Laurie et al., 1982; Simon-Assmann et al., 1986; Beaulieu and Vachon, 1994; Beaulieu, 1997). In the intestine, these molecules are differentially expressed along the crypt–villus axis and are consequently believed to perform distinct roles in IEC processes (Simon-Assmann et al., 1986; Beaulieu, 1997). Another major component of ECMs and BMs is fibronectin, a key player in the establishment and maintenance of tissue morphology and in wound healing (Simon-Assmann et al., 1986; Ruoslahti, 1988; Mao and Schwarzbauer, 2005; Zhang et al., 2006). In the human intestine, fibronectin is strongly expressed in the BM underlying the crypt compartment, decreasing gradually towards the tip of the villus (Quaroni et al., 1978; Simon-Assmann et al., 1986; Beaulieu et al., 1991). Fibronectin is produced, secreted, and deposited by intestinal cells of both epithelial and mesenchymal origins (Quaroni et al., 1978; Laurie et al., 1982; Vachon et al., 1995) and has been shown to contribute to many cell functions in cells of different origins (Ruoslahti, 1988; Mao and Schwarzbauer, 2005). The successful deposition of fibronectin into the BM relies upon its recognition by specific integrin receptors, which mediate its unfolding in a process known as fibrillogenesis (Mao and Schwarzbauer, 2005). This process exposes specific structural domains within fibronectin which then mediate the formation of the insoluble fibronectin fibrils required for their deposition (Mao and Schwarzbauer, 2005). Incidentally, fibronectin deposition is characterized by the formation of specialized ECM–cell contact structures called fibrillar adhesion points, which are generated through an organized interplay between integrins, cytosolic proteins, and actin cytoskeleton (Mao and Schwarzbauer, 2005). While some reports have implicated ILK in the deposition of fibronectin (Wu et al., 1998; Guo and Wu, 2002; Vouret-Craviari et al., 2004; Assi et al., 2008), and although ILK and PIP complexes are found within fibrillar adhesion points (Guo et al., 2001; Vouret-Craviari et al., 2004), the question remains open as to their roles in fibronectin BM deposition in the human intestine. Similarly, the specific role of ILK in adhesion-mediated cell processes remains to be fully understood in human IECs.

In the present study, we investigated the expression and roles of ILK in human IECs in vivo and in vitro. Herein, we report that the crypt–villus axis expression patterns of ILK, PINCH-1, α-parvin, and β-parvin all follow the BM distribution of fibronectin, that is, being largely predominant in proliferative/undifferentiated crypt IECs. Using an siRNA approach to knockdown ILK, we demonstrated a role for ILK in the maintenance of PIP complexes, as well as in the deposition of fibronectin by IECs, subsequently directly affecting their spreading, migration, and cell-cycle progression. The introduction of an exogenous fibronectin matrix rescued the ILK knockdown phenotype, further emphasizing the importance of ILK in the organization of the intestinal crypt–villus BM, as well as providing a functional basis for its significant roles in human IEC cell processes.

## Materials and Methods

### Materials

Mouse primary antibodies used were directed against: ILK (Western blot (WB): 1/1,000) (clone 3/ILK, BD Transduction Laboratories, Franklin Lakes, NJ), PINCH (WB: 1/1,000, immunofluorescence (IF): 1/50, co-immunoprecipitation (Co-IP): 2 µg/100 mm dish) (clone 49/PINCH, BD Transduction Laboratories), β-actin (WB: 1/75,000) (clone C4, Santa Cruz Biotechnology, Inc., Santa Cruz, CA), FAK (WB: 1/1,000) (clone 77, BD Transduction Laboratories), fibronectin (WB: 1/500, IF: 1/50) (HFN 7.1, Developmental Studies Hybridoma Bank, Iowa City, IA), vinculin (IF: 1/500) (clone 7F9, Chemicon International, Temecula, CA), pRB (WB: 1/1,000) (clone G3-245, BD Pharmingen, San Diego, CA), V5 tag (IF: 1/500) (Invitrogen, Eugene, OR), α-parvin (WB: 1/10, IF: 1/2; 3B5; generous gift from C. Wu, University of Pittsburgh), β-parvin (WB: 1/300) (11A5; generous gift from C. Wu), and HA probe (Co-IP, 2 µg/100 mm dish) (Santa Cruz Biotechnology, Inc.). Rabbit primary antibodies used were directed against: laminin (WB: 1/1,000, IF: 1/200) (EHS; Sigma–Aldrich, Oakville, ON), α5 integrin (WB: 1/1,000) (AB1928, Millipore, Temecula, CA), αV integrin (WB: 1/1,000) (AB1930, Millipore), p21 (WB: 1/2,000) (Santa Cruz Biotechnology, Inc.), p27 (WB: 1/2,000) (Santa Cruz Biotechnology, Inc.), and cyclin D1 (WB: 1/5,000) (Santa Cruz Biotechnology, Inc.). Secondary antibodies used were Alexa Fluor 647 goat anti-mouse (Invitrogen) and sheep anti-mouse rhodamine conjugate, sheep anti-rabbit FITC conjugate, and TRITC-conjugated phalloidin (Chemicon International). All other materials were purchased from Sigma–Aldrich, MP Biomedicals (Aurora, OH), BD Biosciences (San Jose, CA), or Fisher Scientific (St. Laurent, QC, Canada) except where otherwise specified.

### Human IEC models and cell culture

Human intestinal epithelial cells (HIECs), which exhibit all the morphological and functional characteristics of normal human proliferative/undifferentiated crypt IECs, were generated from the mid-gestation (18–20 weeks) human ileum and have been characterized elsewhere (Perreault and Beaulieu, 1996). The HIEC line Caco-2/15, a stable clone of the parental Caco-2 cell line (ATCC, Manassas, VA), has been fully characterized in previous studies (Beaulieu and Quaroni, 1991; Pageot et al., 2000). Caco-2/15 cells undergo a full enterocytic differentiation process that takes place spontaneously upon reaching confluence (0 days post-confluence; PC) and which is gradually completed by 25–30 days PC. Differentiated Caco-2/15 cells exhibit all the morphological and functional characteristics of mature enterocytes including their transcriptome (Pageot et al., 2000; Tremblay et al., 2006). HIEC and Caco-2/15 cells were routinely cultured in plastic dishes (100 mm; Falcon Plastics, Los Angeles, CA), and maintained as previously described (Teller et al., 2007).

### RNA interference, transfection, and transduction

An siRNA against ILK (siILK) was purchased from Ambion (Austin, TX). A non-silencing negative control siRNA (siCNS) was purchased from Qiagen (Mississauga, ON, Canada). One day prior to transfection, 2 × 10^5^ cells were plated in 35 mm dishes (Falcon Plastics) and transfected with siRNAs (40 µM final concentration) using the X-tremeGENE siRNA transfection reagent (Roche Diagnostics, Laval, QC, Canada) for HIEC or HiPerfect (Qiagen) for Caco-2/15 cells according to the manufacturers' instructions. Cells were considered ready to be used in the various experiments 48 h post-transfection.

Alternately, HIEC cells were stably transduced with a cDNA coding for a V5-tagged wtILK (a generous gift from S. Dedhar, University of British Columbia, BC). The establishment of stably transduced cell populations was performed according to a retroviral strategy previously described (Escaffit et al., 2006).

### Cell culture-deposited ECM preparations

Newly confluent HIEC cells on plastic culture dishes were lysed using a 27 µM ammonium hydroxide (NH_4_OH) solution for 5 min at room temperature followed by incubation for 10 min with doubled distilled water under rotation to remove remaining cell debris. The underlying insoluble matrix was recuperated in 1× Laemmli buffer (2.3% SDS, 10% glycerol, 0.001% bromophenol blue in 62.5 mM Tris–HCl, pH 6.8).

### Exogenous fibronectin assays

Human plasma fibronectin (Chemicon International) in PBS (pH 7.4) was coated onto either culture dishes or glass coverslips (12 mm diameter) at 3 µg/cm^2^ for 2 h at 37°C. Any remaining potential adhesion sites were blocked with 2% bovine serum albumin (BSA)–PBS (pH 7.4) for 1 h at 37°C prior to plating cells.

### Cell proliferation assays

Proliferation assays were performed using 5-bromo-2-deoxyuridine (BrdU) incorporation. For each assay, 2 × 10^5^ siRNA-transfected HIEC or Caco-2/15 cells were seeded into 35 mm dishes and allowed to settle for 24 h under normal culture conditions. BrdU (10 µM final concentration) (Roche Diagnostics) was added to the medium for 4 h. Visualization of BrdU-positive cells was carried out according to the manufacturer's instructions, and nuclei were stained with 10 ng/ml of 4′,6-diamidino-2-phenylindole (DAPI). Preparations were viewed with a DMIRBE microscope (Leica, Saint-Laurent, QC, Canada) equipped for epifluorescence and digital imaging (RTE/CCD Y/Hz-1300 cooled camera, Princeton Instruments, Inc., Trenton, NJ). The proliferation index was established by calculating the ratio of positive BrdU-stained cells over the total number of DAPI-stained cells × 100.

### Human intestinal tissue samples

Mid-gestation (20 weeks) human small intestine (ileum) specimens were obtained from normal elective pregnancy terminations. Adult human small intestine (ileum) specimens were obtained from non-diseased tissue (at least 10 cm from lesions) of resected ileum from non-inflammatory pathologies (bowel obstruction, primary lymphoma, or tumor). In all cases, only specimens obtained rapidly (60 min or less) were used. For some analyses, mid-gestation ileum specimens were processed for the separation of the epithelium from its underlying mesenchyme, as previously described (Perreault et al., 1998). The project was in accordance with a protocol approved by the Institutional Human Research Review Committee of the Université de Sherbrooke for the use of human material for research.

### Indirect immunofluorescence

The preparation and embedding of tissue samples for cryosectioning were performed as described previously (Beaulieu, 1992). Cryosections (3-µm thick) were prepared, fixed, and stained for indirect IF as previously described (Beaulieu, 1992; Ni et al., 2005). Both primary and secondary antibodies were diluted in PBS (pH 7.4) containing 5% non-fat powdered milk (BLOTTO) or 2% BSA. Some sections were counterstained with 0.01% Evan's blue in PBS (pH 7.4), mounted in glycerol–PBS (9:1) containing 0.1% phenylenediamine and viewed with a Reichert Polyvar 2 microscope (Leica) equipped for epifluorescence and digital imaging (DFC300FX camera; Leica). Cells plated on glass coverslips (12 mm) were prepared and processed for indirect IF as previously described (Vachon and Beaulieu, 1995). Both primary and secondary antibodies were diluted in PBS (pH 7.4) containing 5% BLOTTO or 2% BSA. Nuclei were counterstained 2 min at room temperature with 10 ng/ml DAPI–PBS (pH 7.4), samples were mounted in glycerol–PBS (9:1) containing 0.1% phenylenediamine and viewed with a DMRXA microscope (Leica) equipped for epifluorescence and digital imaging (RTE/CCD Y/Hz-1300 cooled camera).

### Protein complex co-immunoprecipitation

Cells were washed with ice-cold PBS and solubilized in ice-cold lysis buffer (10 mM Tris–HCl, pH 7.4, 150 mM NaCl, 1 mM EDTA, 1% Triton X-100, and Complete Mini, EDTA-free protease inhibitor; Roche Diagnostics) for 20 min on ice, and then centrifuged for 15 min at 13,000*g*. Samples were pre-cleared using protein G-Sepharose (Invitrogen) for 1 h at 4°C. Primary antibodies were added to the samples and incubated overnight at 4°C followed by the addition of protein G-Sepharose for 1 h at 4°C. Samples were washed three times with lysis buffer and resuspended in 2× Laemmli, for subsequent Western blotting analyses (see below).

### Western blotting analyses

Cell cultures were lysed in 1× Laemmli, reduced with 5% β-mercaptoethanol, and processed as previously described (Ni et al., 2005). Total proteins (100 µg/lane) were resolved by SDS–PAGE on 12–15% gels, electrotransferred onto nitrocellulose membranes (BioRad, Mississauga, ON) and probed as described previously (Ni et al., 2005). Full-range molecular mass markers (Full-Range Rainbow Markers, GE Healthcare, Baie D'Urfé, QC, Canada) were used as standards. Immunoreactive bands were visualized using the enhanced chemiluminescence (ECL) method (GE Healthcare) according to the manufacturer's instructions.

### Reverse transcriptase (RT)-polymerase chain reaction (PCR) analyses

Total RNA was extracted using the TriPure isolation reagent (Roche Diagnostics) according to the manufacturer's instructions. RT-PCR was performed as described previously (Ni et al., 2005). Primers used were: for ILK, ILK-F: 5′-AAG GTG CTG AAG GTT CGA GA and ILK-R: 5′-ATA CGG CAT CCA GTG TGT GA; for α-parvin, α-parvin1: 5′-CAA TTC GAC TCC CAG ACC AT, and α-parvin2: 5′-TGG TCG AAC AAG GTG TCA AA; for β-parvin, β-parvin1: 5′-AGG TCC TCC TCG ACT GGA TT, and β-parvin2: 5′-ACC GTC TGC AGC TTC TGT TT; for PINCH-1, PINCH1-F: 5′-TCC CAA GCC CTG ATA ACA AC, and PINCH1-R: 5′-GGG CAA AGA GCA TCT GAA AG; for vimentin, vimentin-F: 5′-AGA TGG CCC TTG ACA TTG AG, and vimentin-R: 5′-GGT CAT CGT GAT GCT GAG AA; for E-cadherin, Ecad-1: 5′-CCT TCC TCC CAA TAC ATC TCC C, and Ecad-2: 5′-TCT CCG CCT CCT TCT TCA TC; for RPLPO (ribosomal protein, large, P0), RPLPO-F: 5′-GCA ATG TTG CCA GTG TCT G, and RPLPO-R: 5′-GCC TTG ACC TTT TCA GCA A. Each cycle was composed of template denaturation at 94°C for 45 sec, primer annealing at 60°C for 45 sec, and elongation at 72°C for 1 min for 30 cycles.

### Real-time PCR quantification analyses

For the quantitative evaluation of RNA messenger levels, real-time PCR (qPCR) was carried out as previously described (Ni et al., 2005). The primers used for ILK and RPLPO were the same as above. Other primers used were: for fibronectin, FN-F: 5′-GTT GTT ACC GTG GGC AAC TC, and FN-R: 5′-CTG ACG GTC CCA CTT CTC TC; for laminin β1chain, LAMB1-up: 5′-GGA ACA GCT CTC CAA GAT GG, and LAMB1-down: 5′-CTG CTT CAA TGC TGT CCA AA; for dipeptidylpeptidase IV (DPPIV), DPPIV-F: 5′-AAG TGG CGT GTT CAA GTG TG, and DPPIV-R: 5′-CAG GGC TTT GGA GAT CTG AG; for villin, Vil1-F: 5′-GGC CAG CCA AGA TGA AAT TA, and Vil1-R: 5′-CTC AAA GGC CTT TGG TGG TGT; and for sucrase–isomaltase (SI), SI-S: 5′-GAG GAC ACT GGC TTG GAG AC, and SI-A: 5′-ATC CAG CGG GTA CAG AGA TG. Amplification efficiencies and assessment of differences in gene expression between controls and experimental conditions were established according to the Pfaffl mathematical model (Pfaffl, 2001). Relative mRNA expression levels were established by comparing the levels from experimentals to those from controls × 100 (expressed as “% of control”).

### Migration and restitution assays

Twenty-four hours prior to wounding both HIEC and Caco-2/15 cells were treated with 2 mM hydroxyurea in order to prevent proliferation while leaving migration unaffected (Hamuro et al., 2002). For HIEC, wound-healing assays were performed as previously described (Dignass and Podolsky, 1993). The relative migratory capacity was established by comparing the total number of cells having migrated across the wound border in treated cultures with that of controls × 100 (expressed as “% of control”). For spreading assays, cells were plated onto glass coverslips (see above) and processed for detection of vinculin by indirect IF to monitor cell spreading after 4 and 18 h (see above). Caco-2/15 siRNA-treated cells were plated at high density and grown until confluence (0 days PC) in 35 mm dishes and monolayers were wounded by aspiration with a 200-µL pipette tip (0.5–0.6 mm diameter) attached to a vacuum source. In all cases, wounds were immediately rinsed and replenished with fresh complete media, complemented or not with 3 µg/cm^2^ of exogenous fibronectin (Chemicon International). Wound areas were measured numerically using the MetaMorph Imaging System (Universal Imaging Corp., West Chester, PA) immediately following and 48 h after wounding. The results were expressed as a percentage of the restitution of the original wounded area size. Only wounds comparable in size and form were used in the statistical analyses.

### Cell aggregate assays

Cell aggregate assays were established in order to simultaneously evaluate cell spreading and migration abilities. Cells were treated with 2 mM hydroxyurea in order to prevent proliferation while leaving migration unaffected (Hamuro et al., 2002). Cells from cultures were detached with a 0.5 M EDTA aqueous solution (15 min at 37°C), centrifuged 4 min at 1,000*g* at room temperature, and resuspended in 5 ml of normal culture medium. Resuspended cells were then transferred to 100 mm polystyrene Petri dishes previously blocked with a 2% BSA–PBS (pH 7.4) solution and incubated 4 h at 37°C, with gently shaking every 30 min. Cell aggregates were then transferred to plastic culture dishes, previously coated or not with fibronectin, and further incubated 1 h at 37°C. After this time, cell aggregates of approximately 50–100 cells were observed with an inverted microscope (M40-82797, Wild Heerbrugg, Leica Canada, Toronto) and marked. After a further 24 h, micrographs of the identified aggregates were taken with the inverted microscope's digital camera (RS Photometrics, CoolSNAP, Tucson, AZ) and the area covered by the spread/migrated cells was subsequently measured numerically using the MetaMorph Imaging System (Universal Imaging Corp.). The relative measure of spreading and migration was established by calculating the ratios of the area covered by cells from the aggregates over the total number of cells in the spread aggregate compared to controls × 100 (expressed as “% of control”).

## Results

### In vivo and in vitro expression of ILK and PIP complex components in human IECs

**Fig. 1:**
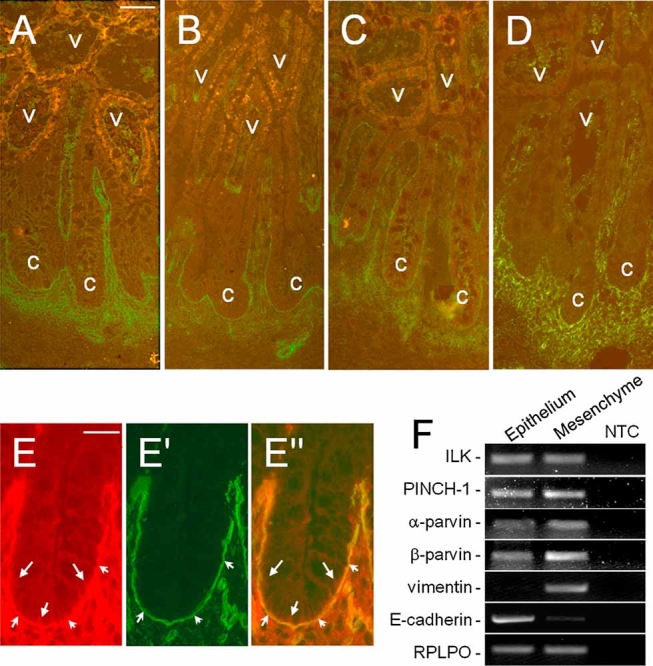
Expression of ILK, PINCH1, α-parvin, and fibronectin in the human small intestine. Immunolocalization on human intestinal cryosections of (A) ILK, (B) PINCH-1, (C) α-parvin, and (D) fibronectin with specific antibodies showed strong staining in the basal membrane of the epithelial and mesenchymal compartments along the crypt. Parts A–D are a composite of adjacent areas. Immunolocalization of (E) ILK and (E′) the laminin chains alone (small arrows) or (E′′) together confirmed the localization of ILK at the epithelial basal membrane (large arrows). F: Semi-quantitative RT-PCR of ILK, PINCH-1, α-parvin, and β-parvin on intestinal epithelial and mesenchymal fractions showed epithelial and mesenchymal expression for each protein. Vimentin and E-cadherin were used to control for fraction purity, and RPLPO was used as a normalizing gene. NTC, no template control; c, crypts; v, upper half of the villus. A–D: Scale bar in (A): 50 µm; in (E–E″), scale bar in (E): 20 µm.

We first sought to establish the in vivo expression patterns for ILK and other components of the PIP complex in the human small intestine. In the functional ileum, ILK was found to be localized at the basal plasma membrane of the intestinal epithelium along the entire length of the crypt–villus axis, but in a clear expression gradient with stronger ILK staining in the crypt compartment than in the villus (Fig. 1A). Components of the PIP complex, namely PINCH-1 (Fig. 1B), α-parvin (Fig. 1C), and β-parvin (data not shown) were also found to be expressed in association with the basal plasma membrane of the intestinal epithelium, and all displayed highly similar patterns of decreasing expression along the crypt–villus axis as observed for ILK. The antibody used to detect PINCH recognizes both PINCH-1 and PINCH-2; however, it has been shown elsewhere that PINCH-2 is not expressed in the intestinal epithelium and mesenchymal cells (Braun et al., 2003). Epithelial localization for ILK was confirmed by co-detection of ILK with a BM component, the laminin β1γ1-chain, allowing for a clear discrimination between the epithelial and underlying mesenchymal populations of the crypt–villus axis (Fig. 1E–E″). ILK was also found to be widely expressed throughout the mesenchymal elements including smooth muscle and endothelial cells (Fig. 1A,E). The in vivo intestinal expression patterns of ILK, PINCH-1, α-parvin, and β-parvin were noted to closely follow the previously established decreasing gradient of fibronectin BM deposition along the crypt–villus axis (Quaroni et al., 1978; Simon-Assmann et al., 1986; Beaulieu et al., 1991; Fig. 1D).

We next confirmed the in vivo expression of ILK and P1IP complex components in IECs by RT-PCR analyses of intestinal epithelium fractions that had been separated from the underlying mesenchyme. Indeed, ILK, PINCH-1, α-parvin, and β-parvin mRNAs were found to be readily expressed in isolated IECs (Fig. 1F). Epithelial and mesenchymal cell fraction purity was confirmed by monitoring the expression of vimentin, a mesenchymal intermediate filament protein, and E-cadherin, an epithelial cell–cell junction protein (Fig. 1F). The expression of RPLPO mRNA was used for normalization (Fig. 1F).

**Fig. 2:**
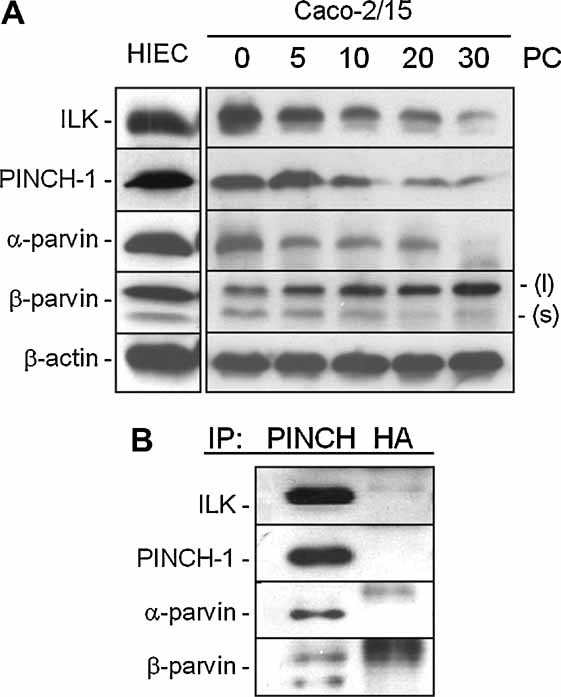
Regulation of P1IP complex members during proliferation in differentiating intestinal epithelial cells. A: Representative Western blot analyses of whole cell lysates of HIEC and Caco-2/15 cells at different stages of post-confluence (0–30 days) using specific antibodies directed against members of the P1IP complex. ILK, PINCH-1, α-parvin, and β-parvin were expressed in our in vitro models. PINCH-1, ILK, α-parvin, and β-parvin(s) (lower band) expression decreased during Caco-2/15 differentiation. β-parvin(l) (upper band) increased during the differentiation process. β-actin was used as a loading control. Representative Western analysis of experiments carried out at least three times independently. B: Co-IP of the P1IP members using PINCH antibody and HA-probe antibody as control on HIEC cells. Immunoblot analysis shows co-immunoprecipitation of ILK, α-parvin, and two isoforms of β-parvin with PINCH-1. Representative Western analysis of experiments carried out three times independently. PC, post-confluence.

Protein expression of ILK and P1IP complex components was analyzed by WB in two human IEC in vitro cell models, HIEC and Caco-2/15. The crypt-like HIEC cells strongly expressed ILK, PINCH-1, and α-parvin (Fig. 2A). These proteins were all detected in Caco-2/15 cells and interestingly, ILK, PINCH-1, and α-parvin were found to decrease as a function of enterocytic differentiation (ILK, 68.9 ± 6.6%; PINCH-1, 70.7 ± 10.0%; and α-parvin, 63.9 ± 7.2% decrease compared to 0 day PC values, n ≥ 3) (Fig. 2A), thus mirroring their observed crypt-to-villus gradients of expression in vivo. The two ubiquitously expressed protein forms of β-parvin, β-parvin(l) and β-parvin(s) (Sepulveda and Wu, 2006), were also detected in both HIEC and Caco-2/15 cells; however, the (l) isoform was clearly predominant (Fig. 2A). Furthermore, the expression of these two β-parvin isoforms appeared to be distinctly modulated as a function of enterocytic differentiation, where β-parvin(l) levels increased and β-parvin(s) levels decreased (β-parvin (l): 260.1 ± 64.0% increase and β-parvin(s): 62.0 ± 8.8% decrease compared to 0 day PC values, n ≥ 3) (Fig. 2A). The expression of β-actin was used as a loading control (Fig. 2A). HIEC cell extracts were used to confirm the organization of these proteins as a complex. A PINCH antibody was used for immunoprecipitation and immunoblot analyses of the IP showed that all members of the P1IP complex co-immunoprecipitated with PINCH-1 (Fig. 2B). The mouse monoclonal anti-HA was used as the IP control antibody because this antibody does not recognize an antigen in HIEC and is the same subtype as the PINCH-1 antibody (IgG_2a_; Fig. 2B).

**Fig. 3:**
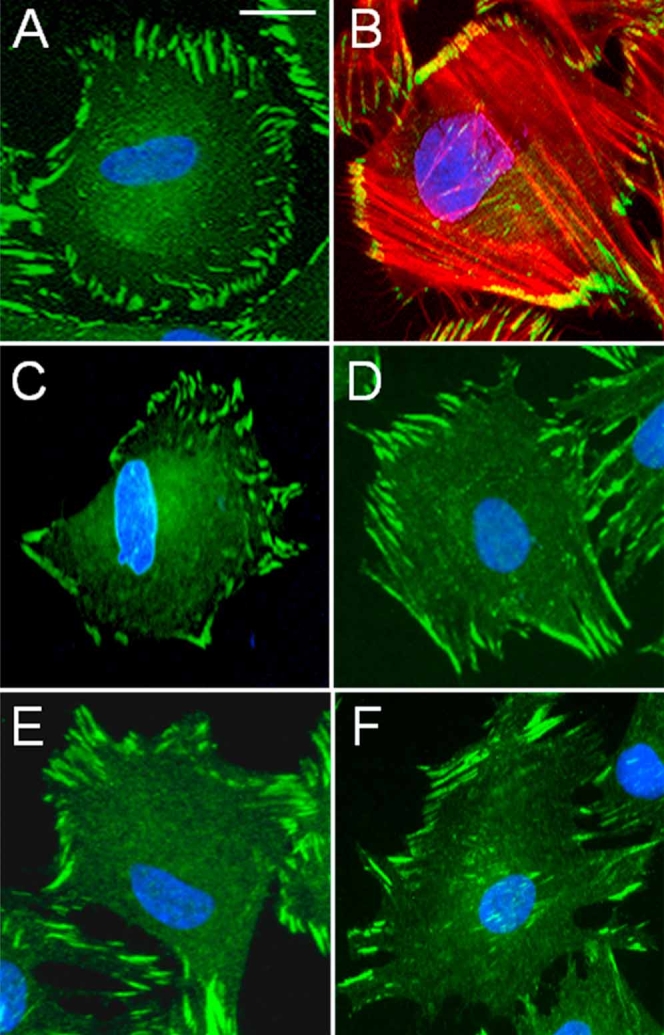
Localization of P1IP members at focal adhesion sites in HIEC cells. Cells were plated on glass coverslips, fixed, and stained with different specific antibodies against (A) vinculin (green) or (B) vinculin (green) co-localized with F-actin (red), (C) V5-ILK, (D) PINCH-1, (E) α-parvin, and (F) β-parvin. Staining patterns were typical of focal adhesion points. A–F: Scale bar in (A): 10 µm. Representative images of experiments carried out at least three times independently.

Indirect IF assays in HIEC cells stably transduced with a cDNA coding for a V5-tagged wtILK were performed in order to verify that ILK and P1IP complex components were associated with focal adhesion points in adherent crypt IECs. ILK was found to be predominantly located at the periphery of the cells in a discrete punctuated pattern typical of focal adhesion points (Fig. 3C), as observed with the focal adhesion point component vinculin (Fig. 3A). Co-staining of vinculin with the actin cytoskeleton showed that focal adhesion points in HIEC were correctly positioned at the extremities of the actin stress fibers (Fig. 3B). PINCH-1, α-parvin, and β-parvin (Fig. 3D–F) displayed intracellular localization patterns highly similar to ILK and vinculin (Fig. 3A,C). These results correlated with our in vivo observations that P1IP complex components were found at the basal plasma membrane in human IECs.

Taken together, these data indicate that ILK is predominantly expressed in human proliferative/undifferentiated IECs, and most likely mediates formation of a PINCH-1–ILK–parvin (P1IP) complex in addition to revealing a relationship between the decreasing crypt-to-villus gradient of expression of this P1IP complex and that of BM deposition of fibronectin. These in turn suggest a possible implication of the P1IP complex in cell adhesion-mediated crypt IEC processes.

### ILK knockdown leads to a severe disruption of the P1IP complex in human crypt IECs

**Fig. 4:**
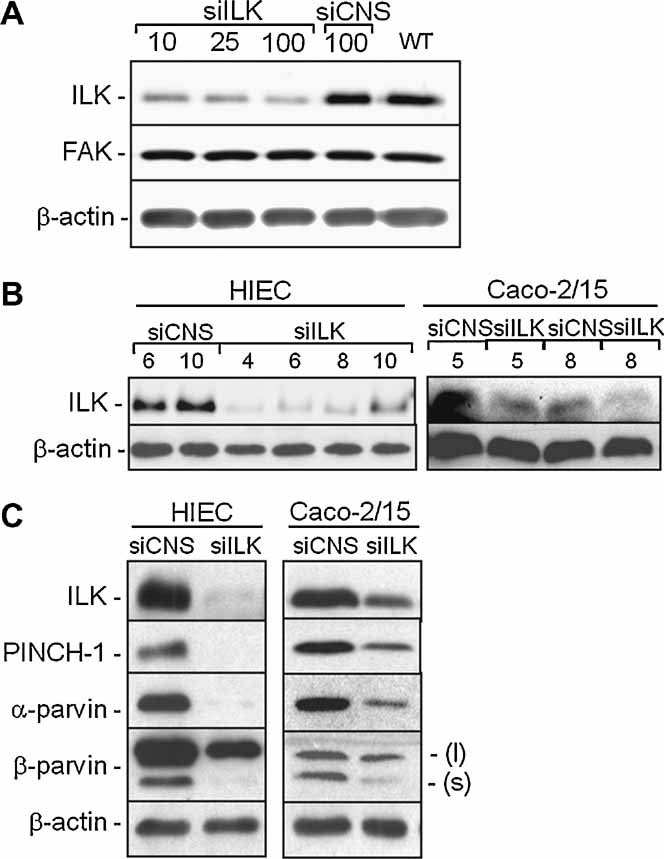
siRNA inhibition of ILK expression in IEC cells. A: HIEC cells were transfected with 10, 25, or 100 µM of siRNA directed against ILK (siILK), 100 µM of a non-silencing negative control siRNA (siCNS), or without siRNA (WT) and monitored for ILK expression. Western analysis for FAK and β-actin protein levels was used as controls for siRNA specificity and loading, respectively. B: HIEC and Caco-2/15 cells transfected with 40 µM of siILK or the non-silencing negative control siCNS were analyzed by Western blot for ILK expression as a function of time (HIEC, 4–10 days; Caco-2/15, 5 and 8 days). β-actin was used as a loading control. C: HIEC and Caco-2/15 cells were transfected with 40 µM siILK, harvested after 72 h (Caco-2/15 cells were at 0 days PC), and analyzed by Western blot using specific antibodies against ILK, PINCH-1, α-parvin, and β-parvin. β-actin was used as a loading control. siILK caused a decrease in expression of all P1IP components in both cell types. Representative Western analysis of experiments carried out at least three times independently. WT, wild type.

An siRNA approach was chosen to determine the functional role of ILK in IECs: First, in undifferentiated/proliferative HIEC cells, a state which appeared to express the highest levels of ILK and then, in proliferative to differentiating Caco-2/15 cells. Transient transfection of increasing concentrations (10, 25, and 100 µM) of an siRNA targeted against the human ILK mRNA (siILK) in HIEC cells resulted in a significant decrease in ILK protein expression (Fig. 4A), in sharp contrast with a control siRNA (siCNS) and a transfection control (Fig. 4A). ILK knockdown did not alter the expression levels of focal adhesion kinase, FAK (Fig. 4A), nor did it activate the OAS interferon response gene (data not shown) suggesting that the ILK knockdown was efficient and specific. Further time-course experiments showed that the siRNA knockdown of ILK was maintained over a period of at least 8 days post-transfection in both HIEC and Caco-2/15 cells (Fig. 4B). Since it has been proposed that PINCH and parvins form obligate complexes with ILK in order to avoid proteasome degradation (Fukuda et al., 2003a), we evaluated the impact of ILK knockdown on the expression of these components in HIEC and Caco-2/15 cells. In siILK cells a drastic loss of PINCH-1, α-parvin, and β-parvin expression resulted from the knockdown of ILK (Fig. 4C). Furthermore, as observed during the Caco-2/15 differentiation process (Fig. 2A), β-parvin(s) is the only β-parvin isoform negatively affected by the reduction of ILK levels (Fig. 4C), suggesting that contrary to β-parvin(l), β-parvin(s) most likely depends on P1IP complex formation to maintain its presence in differentiating IECs. Therefore, these data indicate that the specific knockdown of ILK in human IECs led to a severe disruption of the P1IP complex in these cells.

### ILK knockdown in human crypt IECs leads to a decrease in fibronectin deposition

**Fig. 5:**
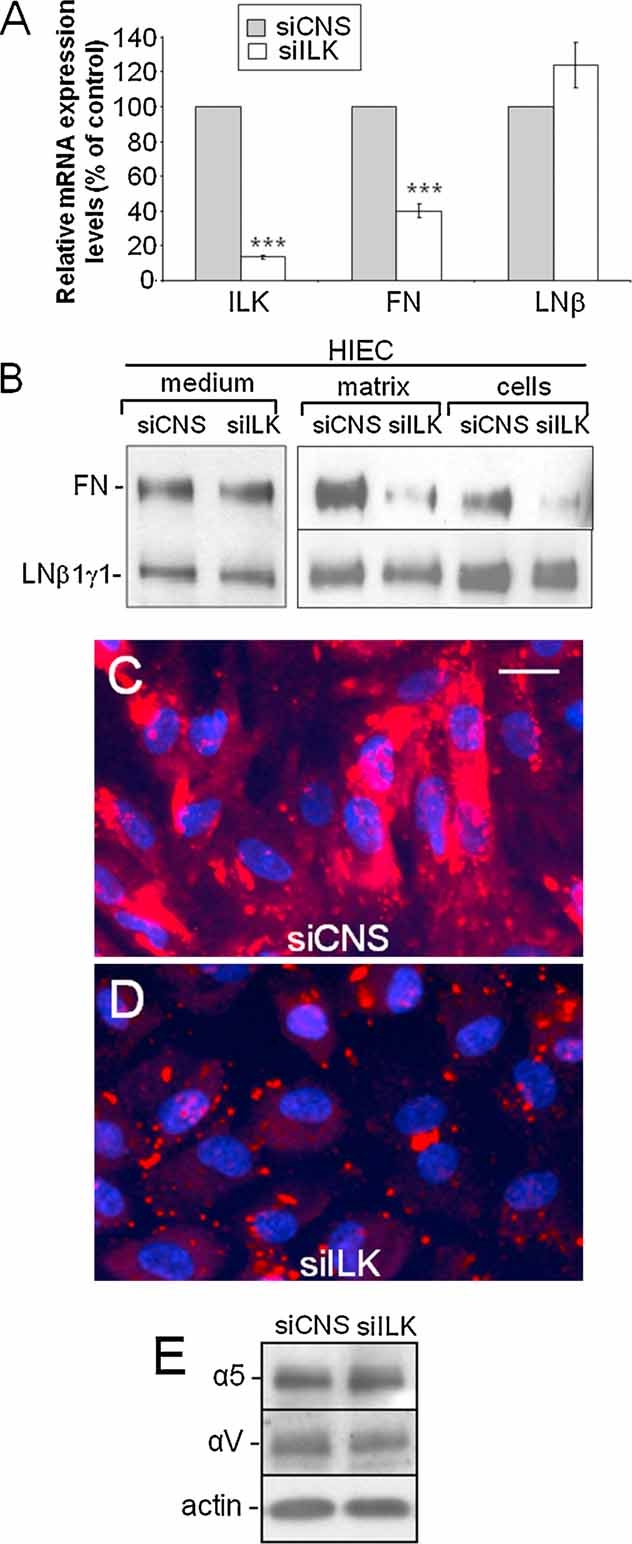
Knockdown of ILK expression inhibits IEC cell fibronectin deposition. HIEC cells were transfected with 40 µM of siCNS or siILK. A: Real-time PCR was carried out on HIEC cells to measure levels of ILK, fibronectin (FN), and laminin β1-chain (LNβ) mRNA levels in siCNS-transfected cells (gray columns) versus silk-transfected cells (white columns). siILK cells showed a significant decrease in ILK and fibronectin mRNA levels. Results are expressed as mean ± standard error of the mean (SEM) and are from three independent experiments. ***P < 0.001 in a one-sample *t*-test with the hypothetical mean of 100 for the normalized values of siCNS. B: Protein levels of fibronectin and the laminin β1γ1-chains were analyzed in the medium, in the insoluble matrix, and in whole cell lysates of HIEC siCNS and siILK cells, and a decrease of fibronectin levels was observed in siILK cell insoluble matrix and whole cell lysates. Representative Western images from five independent experiments. Indirect immunofluorescence of insoluble fibronectin on HIEC (C) siCNS and (D) siILK cells. C,D: Scale bar in (C): 10 µm. E: Representative Western blot analysis of the α5 subunit of the α5β1 integrin and αV subunit of the αV-containing integrins from siCNS- and siILK-treated HIEC showing no significant variation in three separate experiments. [Color figure can be viewed in the online issue, which is available at www.interscience.wiley.com.]

Previous reports have implicated ILK in the deposition of fibronectin (Wu et al., 1998; Guo and Wu, 2002; Vouret-Craviari et al., 2004; Assi et al., 2008) and we have observed a correlation between the decreasing gradient of expression of P1IP complex components and that of fibronectin BM deposition in vivo. We investigated the impact of ILK knockdown on the ability of human IECs to produce and deposit fibronectin. We first performed qPCR analyses on HIEC cells to quantify and compare fibronectin expression levels between siILK and siCNS cells. We found that fibronectin mRNA transcript levels were significantly lowered following the knockdown of ILK, but not under control conditions (Fig. 5A). This decrease in fibronectin mRNA levels was shown to be specific, as levels of the laminin β1-chain mRNA remained unaffected (Fig. 5A). The same phenomenon was observed at the protein level in siILK-treated HIEC since these cells expressed lower amounts of fibronectin than siCNS-treated cells (39.0 ± 10.3% of siCNS values, n = 5.)

Because fibronectin is first secreted by cells as a compact soluble form before it is deposited in the ECM through fibrillogenesis (Mao and Schwarzbauer, 2005), the levels of soluble fibronectin present in the culture media of treated and non-treated HIEC cells were also determined. Surprisingly, levels of secreted, soluble fibronectin in the culture media of siILK cells remained similar to those detected in siCNS cells for both (106.3 ± 15.5% of siCNS values, n = 5), as was the case for secreted, soluble laminin (Fig. 5B). These results suggest that despite lower cellular fibronectin expression, cells deficient for ILK accumulate comparable levels of fibronectin in the medium than control cells as a consequence of inefficient fibronectin deposition. This prompted us to investigate whether the knockdown of ILK impacted on the actual deposition of fibronectin in human IECs. We monitored the capacity of siILK versus siCNS HIEC cells to form an insoluble fibronectin matrix with the available soluble fibronectin in culture media by isolating deposited ECM fractions from these cultures. We found that ILK knockdown led to a substantial decrease in fibronectin deposition (10.6 ± 3.5% of siCNS values, n = 5) compared to control cells (Fig. 5B). This observed decrease appeared to be specific for fibronectin, since the levels of laminin β1γ1-chains found in the ECM remained unaffected. A significant reduction in fibronectin deposition was also observed in siILK versus siCNS-treated Caco-2/15 (data not shown). Lastly, in situ indirect immunofluorescence assays in non-permeabilized HIEC cell cultures showed less intense and less extensive staining for fibronectin ECM deposition following the knockdown of ILK (Fig. 5D), in comparison to control cells (Fig. 5C). Taken together, these results demonstrate that the knockdown of ILK in human IECs impacted negatively on both the synthesis and the deposition of fibronectin.

Since fibronectin deposition is affected by ILK knockdown in IECs we verified whether the expression levels of the fibronectin integrin receptors α5β1 and αV-containing integrins were affected. WB analyses of the α5 and αV subunit protein levels in HIEC cells showed equal amounts of the α5 and αV integrin subunits between siILK- and siCNS-treated cells (Fig. 5E). This result indicates that ILK knockdown did not affect integrin fibronectin receptor expression.

### ILK knockdown decreases spreading, migration, and restitution of human IECs

**Fig. 6:**
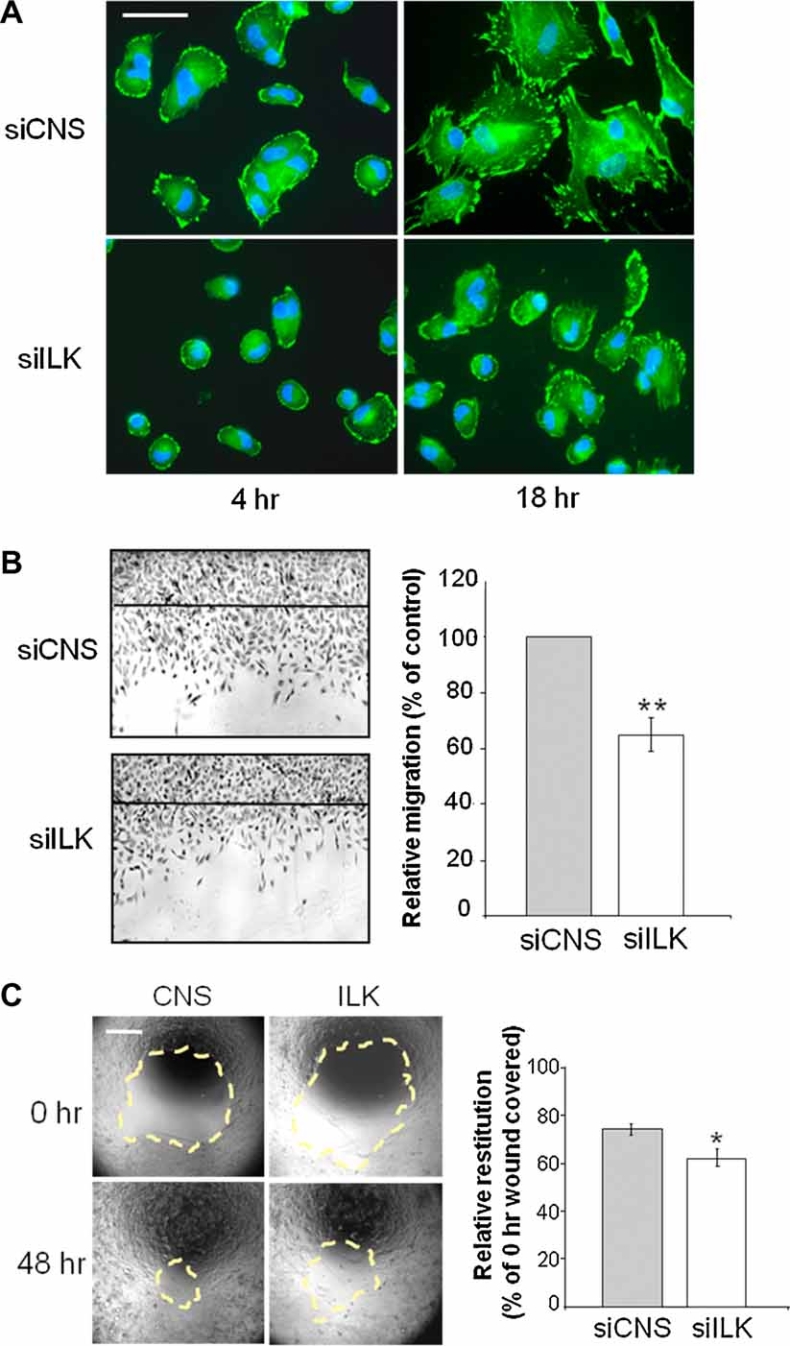
Knockdown of ILK expression inhibits HIEC cell spreading and migration and Caco-2/15 monolayer restitution. HIEC or Caco-2/15 cells were transfected with 40 µM of siCNS or siILK. A: Transfected HIEC cells were plated on plastic and observed at 4 and 18 h, fixed, and stained for vinculin. Scale bar: 20 µm. B: siRNA-treated HIEC were grown on plastic until confluence and wounded with a razor blade. Cells which had migrated into the wounded area after 48 h were counted and expressed as a percentage of control cells. Results are expressed as mean ± standard error of the mean (SEM) and are from three independent experiments. **P < 0.01 in a one-sample *t*-test with the hypothetical mean of 100 for the normalized values of siCNS. C: siRNA-treated Caco-2/15 cells were grown until confluence and the monolayer was wounded by aspiration. Original wound and remaining wound areas where cells had not closed the original wound after 48 h were measured and the results are expressed as a percentage of the restitution of the original wounded area. Results are expressed as mean ± standard error of the mean (SEM) and are from three independent experiments. *P < 0.05 in a paired *t*-test. Scale bar: 100 µm. [Color figure can be viewed in the online issue, which is available at www.interscience.wiley.com.]

Considering that P1IP complex components are associated with focal adhesion points in our cell model, and that the knockdown of ILK resulted not only in a decrease of fibronectin ECM deposition but also in a severe disruption of the P1IP complex, we determined whether ILK knockdown likewise affected the spreading ability and migration capacity of human crypt IECs. siILK- and siCNS-treated HIEC cells were seeded onto glass coverslips and the formation of focal adhesion points by in situ indirect IF staining for vinculin was followed over time. At 4 h post-seeding, both siILK and siCNS cells showed similar rates of adhesion although siILK cells appeared to be less spread than control cells, as visualized by the peripheral localization of vinculin (Fig. 6A). After 18 h, siCNS cells were found to have spread considerably, as evidenced by the extensive display of elongated punctuate patterns of vinculin localization (Fig. 6A) typical of mature focal adhesion points (Geiger et al., 2001). In sharp contrast, siILK cells remained rounded and largely displayed the peripheral dot-like vinculin localization pattern (Fig. 6A) typical of the immature focal adhesion points called focal complexes (Geiger et al., 2001). Hence, these results showed that ILK was not essential for the initial attachment of human crypt IECs but was nonetheless crucial for their subsequent spreading and formation of mature focal adhesion points.

We next verified whether the cell spreading deficiency seen in siILK cells affected crypt IEC migration by performing scratch wound healing assays. As shown in Figure 6B, significantly lower numbers of siILK cells were found to have migrated into the wounded area than siCNS cells. These data indicate that ILK performs a key role in human crypt IEC migration.

A second type of migration found in the polarized intestinal epithelium is restitution, which consists of the resealing of superficial wounds by epithelial sheet movement. The restitution process is a complex phenomenon which does not involve proliferation but requires that the differentiated epithelial cells flatten, spread, and migrate in order to close the wound (Mammen and Matthews, 2003). Since restitution has already been shown to be, at least in part, modulated by the ECM (Zhang et al., 2006) we examined the effect of ILK depletion on Caco-2/15 restitution ability. Results showed that wound closure in newly formed siILK-treated Caco-2/15 cell monolayers was significantly less (62.7 ± 3.0% of the original wound area size) than closure of the wound area in siCNS-treated Caco-2/15 control cells (74.5 ± 3.4%) over the same time period (Fig. 6C). These data point to a role for ILK in the human villus IEC restitution mechanism.

### ILK knockdown leads to decreased proliferation of human IECs

**Fig. 7:**
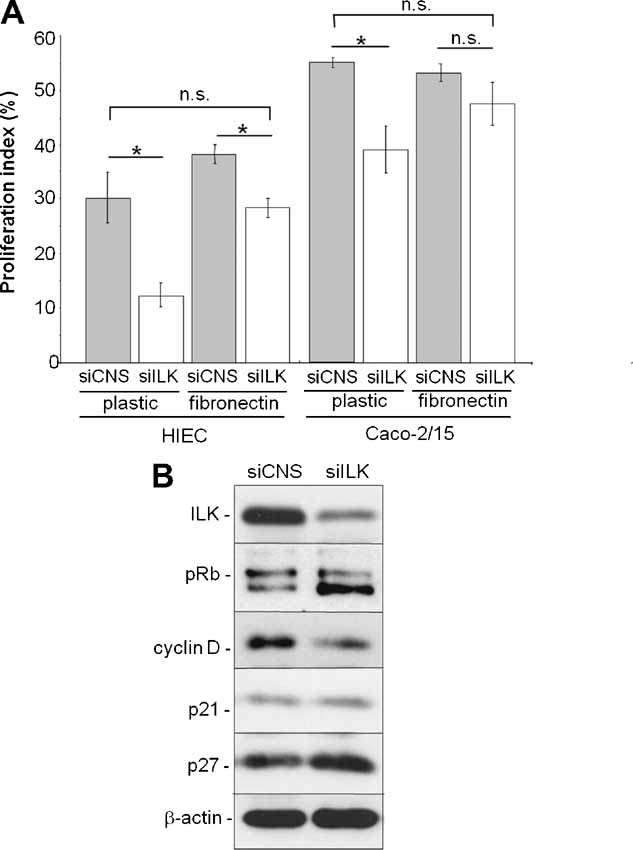
Knockdown of ILK expression inhibits HIEC cell proliferation. HIEC and Caco-2/15 cells were transfected with 40 µM of siCNS or siILK. A: Proliferation indexes were determined by BrdU incorporation on transfected cells that were seeded on plastic or on exogenous fibronectin, and stained using an anti-BrdU antibody. BrdU-positive cells were counted and expressed as a percentage of total cells determined by DAPI staining. Results are expressed as mean ± standard error of the mean (SEM) and are from three independent experiments. *P < 0.05 in a paired *t*-test. B: HIEC-transfected cells were analyzed by Western blot for protein expression levels of ILK, pRB (upper band, hyperphosphorylated form; lower band, hypophosphorylated form), cyclin D, p21, and p27. β-actin was used as a loading control. Representative Western analysis is from at least three independent experiments. n.s., not significant.

Since both ILK and fibronectin have independently been reported to be involved in the control of cell proliferation (Danen and Yamada, 2001; McDonald et al., 2008), we tested whether knockdown of ILK could also negatively impact human IEC proliferation. To this effect, cell proliferation assays (BrdU incorporation) of siILK and siCNS cells cultured on plastic dishes were performed. As shown in Figure 7A, the proliferation index of ILK knockdown HIEC and Caco-2/15 cells was found to be significantly reduced compared to controls. Parallel WB analyses were performed on the normal HIEC cells in order to confirm that this decrease in proliferation was consequent to a deregulation of cell-cycle progression regulatory elements. Indeed, siILK-treated HIEC cells showed sharp increases in the levels of hypophosphorylated pRb and of the cell-cycle inhibitor p27, accompanied by the loss of cyclin D1 levels (Fig. 7B). Levels of expression of the cell-cycle inhibitor p21 remained constant. Therefore, these results indicate that ILK performs crucial functions in the modulation of key regulatory elements of cell-cycle progression of human crypt IECs, thus constituting a molecular basis for ILK's role in stimulating the proliferation of these cells.

We then plated siILK- and siCNS-treated HIEC cells onto exogenously deposited fibronectin and performed additional cell proliferation assays. The proliferation index of siILK-treated HIEC cells was increased when plated on exogenous fibronectin (Fig. 7A), essentially reversing to levels observed for siCNS-treated HIEC cells on plastic (Fig. 7A), although still significantly lower than that of siCNS-treated HIEC cells on fibronectin (Fig. 7A). Similar results were obtained using Caco-2/15 cells. Knockdown of ILK in Caco-2/15 cells significantly reduced the proliferation index in cells grown on plastic (Fig. 7A) whereas it was restored when these cells were plated on fibronectin (Fig. 7A). Interestingly, exogenous fibronectin did not increase the proliferation index of siCNS-treated Caco-2/15 cells (Fig. 7A). These results indicate a key role for ILK in the deposition and organization of fibronectin into a functional matrix by human IECs, which in turn constitutes a major element in driving their proliferation.

### Exogenous fibronectin rescues the ILK knockdown phenotype in human IECs

**Fig. 8:**
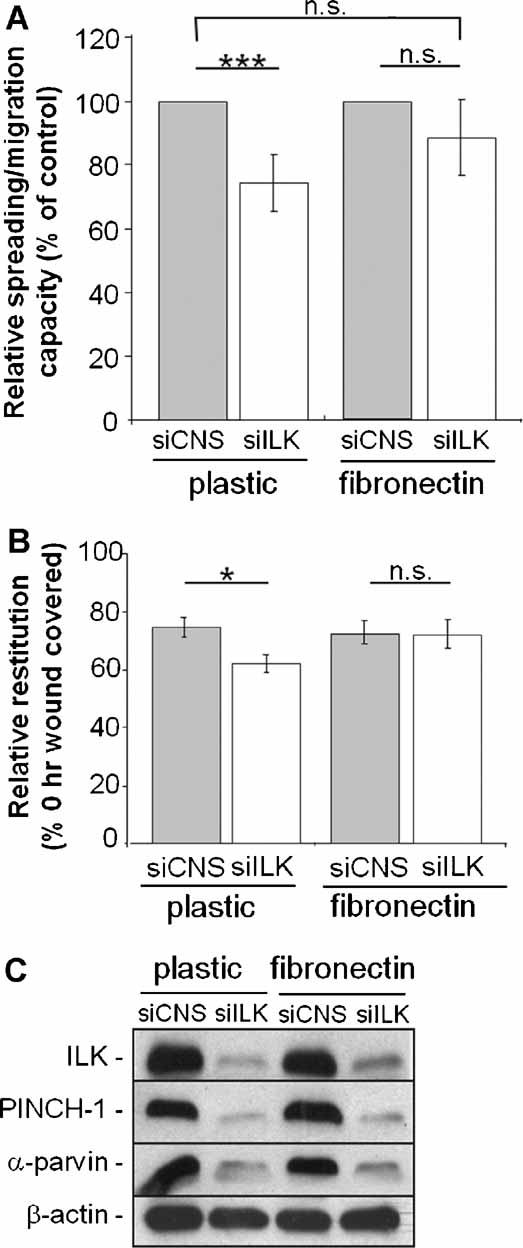
Exogenous fibronectin rescued spreading, migration, and restitution of siILK cells. HIEC or Caco-2/15 cells were transfected with 40 µM of siCNS or siILK. A: HIEC cell aggregates (50–100 cells) were plated on plastic or exogenous fibronectin and analyzed after 24 h. The area covered by the spread/migrated cells was subsequently measured and the relative measure of spreading and migration was established by calculating the ratios of the area covered by cells from the aggregates over the total number of cells in the spread aggregate compared to controls × 100 (expressed as “% of control”). Results are expressed as mean ± standard error of the mean (SEM) and are from at least three independent experiments. ***P < 0.001 in a one-sample *t*-test with the hypothetical mean of 100 for the normalized values of siCNS. B: siRNA-treated Caco-2/15 cells were grown until confluence and the monolayers were wounded by aspiration. Exogenous fibronectin was then added or not to the culture medium. Areas where cells had not covered the original wound after 48 h were measured and expressed as a percentage of the original wounded area. Results are expressed as mean ± standard error of the mean (SEM) and are from at least three independent experiments. *P < 0.05; ***P < 0.001 in a paired *t*-test. C: siILK- and siCNS-treated HIEC cells were plated and grown on plastic or fibronectin for 48 h. The cells were then harvested and analyzed by Western blot. Fibronectin did not rescue the decrease of the PIP components caused by siILK treatment. β-actin was used as a loading control. n.s., not significant.

We then sought to confirm the direct relationship between ILK knockdown, decreased fibronectin deposition, and decreased cell spreading, migration, and restitution. We first performed cell aggregate assays with HIEC cells, which allow for the simultaneous analysis of cell spreading and migration capabilities (Fig. 8A). We also carried out restitution assays with and without fibronectin on Caco-2/15 cell monolayers (Fig. 8B). When on plastic alone, siILK-treated cells exhibited significantly lower spreading/migration and restitution capacities than control siCNS-treated cells (Fig. 8A,B), as expected from our previous results above. The presence of exogenous fibronectin successfully reversed the spreading/migration and restitution reductions observed in siILK cells. Exogenous fibronectin had no net effect on control siCNS cells (Fig. 8A,B). In order to confirm that the previous exogenous fibronectin functional rescue of siILK-treated HIEC does not result from a possible salvage of some P1IP members, we next investigated the individual levels each P1IP member in these cells. WB analyses showed no significant rescue of PINCH-1 nor α-parvin in ILK knocked down HIEC by fibronectin (Fig. 8C). These results indicate that the decrease of fibronectin deposition in siILK cells was directly responsible for the reduction in IEC cell spreading, migration, and restitution.

### ILK knockdown does not influence human IEC differentiation

**Fig. 9:**
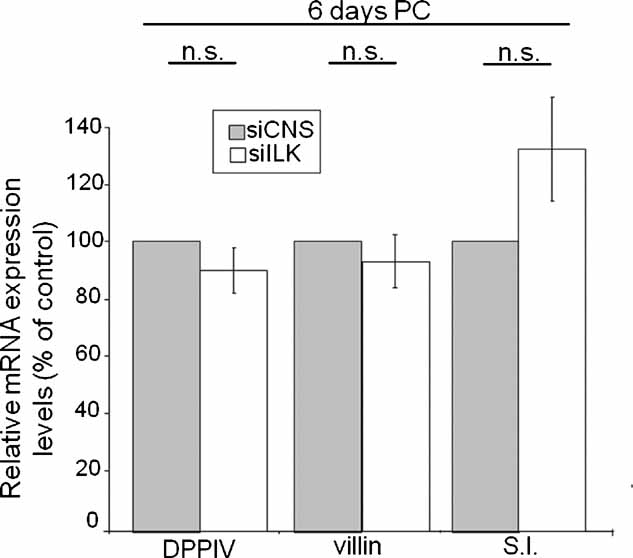
Knockdown of ILK expression does not influence the differentiation process. Caco-2/15 cells were transfected with 40 µM of siCNS or siILK. Real-time PCR was carried out to measure DPPIV, villin, and sucrase–isomaltase (SI) mRNA levels in siCNS cells (gray columns) versus siILK cells (white columns). Abolition of ILK in Caco-2/15 cells did not significantly change DPPIV, villin, and SI transcript levels at 6 days PC. Results are expressed as mean ± SEM and are from three independent experiments. Statistical analysis was one-sample *t*-test with the hypothetical mean of 100 for the normalized values of siCNS. n.s., not significant; PC, post-confluence.

We next verified if the reduction of ILK had an impact on the differentiation of Caco-2/15 cells by monitoring the expression of DPPIV, villin, and SI, all of which are typical markers of intestinal epithelial differentiation (Beaulieu and Quaroni, 1991; Vachon and Beaulieu, 1992; Tremblay et al., 2006). Caco-2/15 cells were infected under the same conditions as in Figure 4B and harvested 6 days after confluence (corresponding to 9 days post-infection, see the Materials and Methods Section for details). Quantitative PCR revealed that the expression of the three tested markers was not altered by the ablation of ILK expression (Fig. 9). This indicates that ILK is not directly implicated in the control of cellular differentiation of human IECs.

## Discussion

In the present study, we investigated the expression and role of ILK in human IECs in vivo and in vitro. Herein, we report that the expression patterns of ILK, PINCH-1, α-parvin, and β-parvin along the crypt–villus axis decreased as a function of enterocytic differentiation and followed the BM distribution of fibronectin, that is, being largely predominant in human proliferative/undifferentiated crypt IECs. In these cells, ILK, PINCH-1, α-parvin, and β-parvin were closely associated with focal adhesion points. The siRNA knockdown of ILK in human IECs caused a severe disruption of P1IP complexes along with a significant decrease in cell proliferation (decrease of cyclin D1, increase in p27 and hypophosphorylated pRb), as well as spreading (persistence of immature focal adhesion points) migration and restitution capabilities. The ILK knockdown-induced defects in spreading, migration, restitution, and proliferation were directly related to a concomitant decrease in fibronectin synthesis and ECM deposition and were reversed with exogenously deposited fibronectin. Hence, ILK performs these key roles in human IECs, most likely as part of a P1IP complex.

A primary function of ILK is to mediate the formation of PIP complexes in a variety of tissues, where the exact PINCH/parvin composition, and consequently their specific roles in cell processes, may be cell and tissue context-dependent (Legate et al., 2006; Sepulveda and Wu, 2006; McDonald et al., 2008). Our findings not only further support the concept of an absolute requirement of the scaffolding function of ILK for the formation and maintenance of active PIP complexes at focal adhesion points but additionally reveal that the levels of the PIP complex may change depending on the state of differentiation within the same given cell type. Indeed, our results showed that proliferative/undifferentiated crypt IECs express high levels of P1IP complex components whereas non-proliferative/differentiated villus IECs express low levels. Our results also suggest that α-parvin and β-parvin(s) have exclusive roles in the context of the PIP complexes in IECs since α-parvin and β-parvin(s), but not β-parvin(l), follow the decline in PINCH-1 and ILK protein levels in differentiating Caco-2/15, as well as in siILK-treated HIEC and Caco-2/15 cells. Consequently, further investigation will be required to fully comprehend the precise roles of the distinct PIP complexes in human IECs.

A key finding in the present study was the demonstration of a direct relationship in human small intestinal cells between the functional expression of ILK and the capacity for fibronectin synthesis and functional matrix deposition. The overexpression of ILK has previously been shown to increase fibronectin ECM deposition in rat IECs (Wu et al., 1998) and the intestine-specific knockout of ILK in mice, as well as the siRNA knockdown of ILK in SW480 colon cancer cells, were recently shown to result in the decrease of fibronectin mRNA and protein expression levels (Assi et al., 2008). Hence, our findings not only corroborate the observations from these previous studies, but furthermore show, for the first time, an implication of the P1IP complex—and not just ILK—in the process of fibronectin synthesis and functional BM deposition in human IECs. Interestingly, expression levels of P1IP members show a decreasing gradient in differentiating Caco-2/15 cells which correlates with the decreasing gradient of fibronectin expression in these cells previously reported by ourselves (Vachon et al., 1995), as well as with our observations in the small intestinal epithelium and BM shown herein. The fibronectin gene promoter contains several response elements (Alonso et al., 1996; Gradl et al., 1999) that have been shown in other cell systems to be potentially regulated by ILK-mediated signaling (Legate et al., 2006; McDonald et al., 2008), thus providing a molecular basis for the relationship between the loss of ILK (and the P1IP complex) and the consequent reduction in fibronectin mRNA transcription. However, this alone cannot account for the observed reduction in fibronectin deposition, since knockdown of ILK did not affect the net amount of fibronectin found in the culture medium. Such apparently contrasting observations are likely the result of the different soluble fibronectin deposition abilities between control and ILK-treated cells. A previous study in rat IECs reported that overexpression of ILK prompted the co-localization of the fibronectin integrin receptor, α5β1, with the focal adhesion point component vinculin (Wu et al., 1998). This observation is consistent with the established requirement of fibronectin integrin receptors for fibrillogenesis and ECM deposition (Mao and Schwarzbauer, 2005). Our findings show that the expression levels of fibronectin receptors are unaffected by the loss of ILK, suggesting that these receptors remain physically available for binding. However, this does not exclude that a scaffolding, and/or a signaling, defect constitutes the driving cause for the observed decrease in fibronectin deposition. Indeed, there is a requirement for actin cytoskeletal tension, generated at focal adhesion points by stress fibers, in enabling fibrillogenesis (Baneyx et al., 2002; Yoneda et al., 2007). Our finding that the knockdown of ILK results in IECs forming immature focal adhesion points suggests that the loss of ILK (and consequently the P1IP complex) greatly reduces the actin cytoskeletal tension so necessary for fibrillogenesis—and consequently impacts negatively on the deposition of fibronectin. Alternatively, various signaling molecules such as PI3-K, Src, and MEK/Erk have also been shown to regulate the deposition of fibronectin in various cell types (Hughes et al., 1997; Brenner et al., 2000; Wierzbicka-Patynowski and Schwarzbauer, 2002). Since these can be regulated by ILK and PIP complexes (Delcommenne et al., 1998; Fukuda et al., 2003b; Kim et al., 2008), their deregulation may also in part explain the reduction of fibronectin deposition that is consequent to a knockdown of ILK. Further studies will be required to gain a better understanding of the molecular basis of the functions of PIP complexes in fibronectin synthesis and ECM deposition, as well as to firmly establish whether the kinase activity of ILK, its scaffolding function, or both, are required in this process. In this respect, a previous study in rat IECs reported the requirement for the integrity of the ILK kinase domain in the up-regulation of fibronectin deposition, following overexpression of ILK (Wu et al., 1998).

Another key finding in the present study is the demonstration of a direct relationship in human crypt IECs between the functional expression of ILK and the capacity for fibronectin matrix deposition, which then regulates spreading, migration, and cell-cycle progression. Fibronectin is well recognized as a key component in the regulation of various cell processes (Wierzbicka-Patynowski and Schwarzbauer, 2003). Its predominant deposition in the BM underlying the crypts correlates with the presence of actively proliferating and migrating IECs in this intestinal epithelial compartment (Pageot et al., 2000). These findings, together with our observation that the differentiation process of siILK-treated Caco-2/15 cells is in no way altered, support previous reports that fibronectin does not affect enterocytic differentiation (Kedinger et al., 1987; Hahn, 1988; Hahn et al., 1990), but rather stimulates IEC spreading, migration, and proliferation (Goke et al., 1996; Kuwada and Li, 2000; Hagerman et al., 2006; Zhang et al., 2006; Assi et al., 2008). It is therefore not surprising that the ILK knockdown-induced deficiency of fibronectin deposition in human crypt IECs was concomitantly accompanied by deficiencies in their spreading, migratory, and proliferative capacities (this study). Furthermore, our results also support a role for a fibronectin/ILK-dependent mechanism in the restitution ability of villus IECs. As we reported herein and elsewhere, mature (non-proliferating/differentiated) villus IECs express low levels of P1IP complex members (this work), as well as low levels of fibronectin (this work) (Vachon and Beaulieu, 1992) compared to their proliferative/undifferentiated crypt counterparts. However, differentiated enterocytes need to dedifferentiate in order to initiate and complete the restitution process (Basson, 2001). Considering that fibronectin has already been shown to promote intestinal restitution (Zhang et al., 2006), it can therefore be expected that dedifferentiated cells present at the wound borders may “re-express” increased levels of P1IP complexes and fibronectin during the restitution process. These data, coupled with our additional observation that exogenously deposited fibronectin rescued the ILK knockdown phenotype, clearly demonstrated a critical regulatory role for ILK and fibronectin in the maintenance of human intestinal crypt–villus axis homeostasis.

Although ILK and PIP complexes have been implicated in various cell processes such as spreading and migration (Sepulveda and Wu, 2006; McDonald et al., 2008), their roles in these processes in IECs have remained poorly understood. We have shown that the loss of ILK (and the P1IP complex) in human crypt IECs not only resulted in defects in the formation/assembly of mature focal adhesion points but also led to significant defects in spreading and migration. However, other focal adhesion components, such as FAK and vinculin, which have been previously shown to be sufficient for spreading and migration (Panetti, 2002; Ziegler et al., 2006), remained unaffected by the knockdown of ILK. This would explain why exogenously deposited fibronectin fully rescued the ILK knockdown phenotype with regard to spreading and migration. In turn, this would also explain why the loss of ILK and of the P1IP complex significantly reduced spreading and migration in human crypt IECs, without managing to fully abrogate these processes. Indeed, while fibronectin is a principal activator of IEC spreading and migration, it is not the only intestinal BM component to exhibit this ability (Goke et al., 1996; Beaulieu, 1997; Teller and Beaulieu, 2001). In any event, much remains to be understood of the molecular contributions of ILK and P1IP complexes in their fibronectin-dependent regulation of spreading and migration in human crypt IECs.

Although proliferation is adhesion dependent in normal cells (Moschos et al., 2007), various elements of this process are distinct to those which mediate spreading and migration (Lock et al., 2008). While ILK and PIP complexes have been implicated in the regulation of cell proliferation (Sepulveda and Wu, 2006; McDonald et al., 2008), their roles in this process in IECs remain to be understood. For instance, whereas a role for ILK in intestinal tumorigenesis is becoming increasingly established (Bravou et al., 2006; Assi et al., 2008), it has only recently been reported in mice that ILK is implicated in the stimulation of normal IEC proliferation and in the maintenance of crypt size (Assi et al., 2008). In the present study, we have shown that ILK and the P1IP complex are predominantly expressed by human crypt IECs in vivo and in vitro and follow the BM deposition pattern of fibronectin along the crypt–villus axis. To this effect, exogenously deposited fibronectin fully restored proliferation in ILK knockdown human crypt IECs. In addition to α5β1, proliferative/undifferentiated (crypt) IECs may also use the integrins αVβ3 and/or αVβ5 as fibronectin receptors (Zhang et al., 2003). The integrin receptor α5β1 engages and activates ILK-mediated signaling upon binding fibronectin (Gopalakrishna et al., 2000; Vouret-Craviari et al., 2004; Camacho-Leal et al., 2007). Furthermore, fibronectin has been previously reported to stimulate ILK activity in rat IECs (Delcommenne et al., 1998), whereas the overexpression of ILK in these same cells increased the expression of cyclin D1 (Radeva et al., 1997). Previous work has reported that ILK (and PIP complexes) can contribute to the activation of signaling pathways that are implicated in the regulation of cell proliferation, such as Src, Akt, GSK-3, and MEK/Erk (Legate et al., 2006; Naska et al., 2006; Kim et al., 2008). The loss of ILK (and of the P1IP complex) in human IECs resulted in a gross deregulation of key regulatory elements of cell-cycle progression (namely pRb, p27, and cyclin D1). Taken together these observations not only strongly support a major role for ILK (and the P1IP complex) in the fibronectin-mediated driving of human crypt IEC proliferation but also begin to provide a molecular basis for such a role. However, those same proliferation-regulating pathways that are susceptible to being activated by ILK and PIP complexes following binding to fibronectin can likewise be engaged by the signaling of various other ECM/BM components and their receptors, as well as by growth factor receptors. It is therefore possible that ILK-deficient IECs are unable to unfold and organize exogenous fibronectin into a fibril matrix, and as a result affect the organization of the ECM/BM itself (Sottile and Hocking, 2002). Consequently, this may explain why exogenously deposited fibronectin fully restored proliferation in ILK knockdown human crypt IECs, yet at the same time failed to further enhance proliferation in these same cells, to levels observed in ILK-expressing/control cells. In turn, this would also explain why the loss of ILK and of the P1IP complex significantly reduced proliferation in human crypt IECs but did not manage to fully abrogate the process. Therefore, additional studies will be required in order to further identify the signaling basis which underlies the role of ILK in the regulation of cell-cycle progression in human crypt IECs.

In conclusion, this study has provided evidence that ILK, and by extension the P1IP complex, performs crucial functions in the control of critical adhesion-mediated cell processes that are required for human intestinal crypt and villus cell homeostasis—namely fibronectin synthesis and BM deposition, as well as cell spreading, migration, restitution, and proliferation. In addition to the present findings, further investigation of these fibronectin-driven, ILK-mediated IEC processes should provide greater knowledge of the complexities involved in the maintenance, renewal, and healing of the human intestinal epithelium—both under normal physiological conditions and within the context of physiopathologies such as inflammatory bowel disease and cancer.
